# From the perceived risks to the preventative actions: analysis of adolescents’ behaviors facing Covid-19*

**DOI:** 10.1590/1980-220X-REEUSP-2025-0389en

**Published:** 2026-04-17

**Authors:** Jayne Ramos Araújo Moura, Mariana Ribeiro Silva, João Rafael da Silva Fonseca, Jaqueline Renata da Silva Brito, Roberta Meneses Oliveira, Ana Larissa Gomes Machado, Neiva Francenely Cunha Vieira

**Affiliations:** 1 Universidade Federal do Ceará, Departamento de Enfermagem, Fortaleza, CE, Brazil.; 2 Universidade Federal do Piauí, Departamento de Enfermagem, Picos, PI, Brazil.; 3 Universidade Federal do Piauí, Departamento de Enfermagem, Teresina, PI, Brazil.

**Keywords:** COVID-19, Coronavirus, Adolescent Behavior, Health Belief Model, Social Isolation.

## Abstract

**Objective:**

To understand adolescents' responses to the Covid-19 pandemic, considering their beliefs and attitudes towards the threat of contagion and transmissibility.

**Method:**

Qualitative study, based on the theoretical framework of the Health Belief Model, conducted in a municipality in northeastern Brazil. Six focus groups were held, with the participation of 45 adolescents. The statements were transcribed, organized into a textual corpus, and submitted to content analysis using IRAMUTEQ software.

**Results:**

Three thematic categories emerged: 1. Perception of risk and susceptibility to Covid-19 – threat, death; 2. Severity and gravity of COVID-19 – isolation, fear; 3. Prevention and protective measures against coronavirus contamination – social distancing, hygiene.

**Conclusion:**

The study showed that the perception of risk and severity of the health crisis contributed to adolescents adopting self-protective behaviors for themselves and their families. Their mental health was affected by feelings of fear and anxiety, reflected in their social interactions during the isolation and post-pandemic periods. It is suggested that there be a greater focus on health actions for this population, as well as studies that identify other outcomes of the period experienced.

## INTRODUCTION

The Covid-19 pandemic period was a crucial moment for understanding how people respond to health crises and learning about the factors that trigger readiness to act individually and collectively in the control and prevention of diseases.

Although not the most vulnerable group during the pandemic, adolescents suffered family losses and were subjected to social isolation that prevented them from interacting with their peers during this stage of transition to adulthood. Studies have demonstrated the psychosocial consequences that affected the growth and development of adolescents, which still remain in the post-pandemic period^(1,2)^.

At the same time, a range of technological learning resources have significantly impacted the daily lives of adolescents, especially in terms of maintaining school activities and communication during the period of social isolation. Although those changes have enabled the continuity of education through digital platforms, the suspension of face-to-face classes and the transition to remote learning have been associated with a significant increase in symptoms of depression and anxiety in that population^(3,4)^.

In addition to the difficulties of following the school curriculum, the absence of the in-person school routine and the limitation of social interactions amplified feelings of loneliness and isolation, negatively impacting the mental health of adolescents^(5)^. The restriction of extracurricular activities and the lack of social spaces have aggravated the emotional vulnerability of young people, resulting in a reduction in the quality of the learning process and productivity and an increase in the prevalence of mental disorders^(6)^.

Despite these challenges, adolescents have shown resilience by seeking alternative ways to maintain socialization and learning through digital tools, connecting with friends and family, and accessing reliable information about the pandemic^(7,8)^.

Studies on adolescent health behavior have generally addressed different risk situations such as unplanned pregnancy, sexually transmitted infections, and alcohol and drug abuse. Evidence has shown that interpersonal relationships have influenced adolescents’ responses to the adoption of preventive measures, including their relationships with their peers^(9,10,11)^.

Furthermore, the National School Health Survey has reiterated regional disparities in terms of health problems affecting adolescents, which calls for the strengthening of educational programs that are more accessible to this population.

The Northeast region had the lowest percentage of mothers with higher education, lower proportions of physically active adolescents, a higher percentage of teenage pregnancies, and a high proportion of less healthy eating patterns when compared to other regions of the country^(11)^.

One of the important aspects highlighted in this study was the fact that the health crisis resulting from the Covid-19 pandemic affected the entire family life cycle and, at the same time, their living conditions, exposing them to situations of coping, such as loss, grief, and economic and social restrictions. The outcomes of this phase may have brought to light elements that interact with other segments of adolescent development, such as coexistence (or absence) in the school environment and interactions with peers that shape their personality and identity in social reality. This reinforces the idea that the educational process for adolescents must dialogue with factors in their environment and emotional bonds, innovating solutions for collective well-being.

Given those facts, nurses, as personnel of health services and systems, especially Primary Health Care (PHC), are an important link in the chain of promoting and protecting adolescent health, mainly in coordinating actions involving other systems, such as schools, performing actions that range from direct care to the production of knowledge that informs clinical practice.

This study, therefore, aimed to understand adolescents’ responses to the Covid-19 pandemic, considering their beliefs and attitudes towards the threat of contagion and transmissibility. The research can contribute to identifying the factors that influence adolescents’ health behaviors in the face of emergency crises, based on the health beliefs model as a theoretical framework.

## METHOD

### Type or Study Design

Qualitative study, developed under the criteria of the Consolidated Criteria for Reporting Qualitative Research (COREQ)^(12)^, based on the theoretical framework of the Health Belief Model (HBM), which allows to understand how the perception of disease risk and belief in the effectiveness of preventive measures directly influence individual behaviors^(13)^.

The HBM helped to understand the reasons why adolescents adhered to preventive measures during the pandemic, such as wearing masks and social distancing, while others did not. In addition, factors such as self-efficacy and internal motivation are relevant for the adoption of practices to prevent and cope with the stress associated with social isolation imposed by the health crisis, which can be better understood from the reference framework.

### Location

The study took place in a municipality located in a state in northeastern Brazil, in a semi-arid region, with an estimated population of 78,002 inhabitants, a human development index (HDI) of 0.698, and severe social inequalities^(14)^, from November 2021 to November 2023.

### Population and Selection Criteria

The study population consisted of adolescents of both sexes, enrolled in the 8th and 9th grades of elementary and high school in public schools from urban areas, aged between 13 and 18 years old.

The inclusion criteria for sample selection were: being enrolled and attending school on the days of data collection; and exclusion criteria were: presenting cognitive limitations that prevent participation in the study, visual and/or hearing impairments that required special educational support to perform school tasks.

### Definition of the Sample

The adolescents were selected through purposive sampling, and quantitative of participants was defined when informational redundancy was reached, by theoretical saturation^(15,16)^.

### Data Collection

For data collection, the Focus Group (FG) technique was adopted^(17)^. The FGs used a script of open-ended questions that sparked discussion, focusing on coping strategies in the face of adversities caused by the Covid-19 pandemic, organized into four stages: (a) introduction of participants; (b) icebreaking activity; (c) trigger questions focused on understanding the perceptions and experiences of adolescents in the Covid-19 pandemic (d) conclusion.

The trigger questions were the following: Is Covid-19 a threat to adolescents? Why is Covid-19 a threat to adolescents’ health? In your opinion, where is Covid-19 the greatest threat and why? Thinking about the entire pandemic period, what were your main fears regarding Covid-19? Why did you believe you could have severe symptoms? What strategies did you adopt to protect yourself from the coronavirus? And, in your opinion, how can adolescents help in the fight against the coronavirus? What were the main difficulties in dealing with the coronavirus? Why? How was the Covid-19 period for you in relation to school, friends, leisure, etc.? How did you feel? What were the consequences of this period for you? What motivates you to take preventive measures against the coronavirus? When you needed it, did you have access to healthcare and/or medication? How was that experience?

The FGs consisted of small/homogeneous groups of between 6 and 10 participants, recorded with specific equipment for approximately one hour.

### Data Analysis and Processing

The generated data were organized and processed as follows: full transcription, preparation of the inventory, which consists of isolating the elements present in the adolescents’ statements and expressions, and generation of the textual corpus.

At this stage of building de textual corpus, the Reflective Content Analysis was used. It is a transtheoretical and flexible method oriented towards the description and reduction of manifest qualitative data. This technique is used to identify patterns in the explicit surface meanings of qualitative data through the application of a hierarchical structure of quantifiable analytical strata called codes, subcategories, and categories. Each stratum exists on a continuum of abstraction, with codes being closest to the original data and categories being the most abstract. During each stage of the process, reflexivity is considered a valuable analytical resource that is crucial to ensuring an adequate description of the data. The technique is intended to be used as a method of data analysis, not a methodology; therefore, it can be integrated with various methodological and epistemological approaches^(18)^.

After this organization, the textual corpus was submitted to analysis in the IRAMUTEQ (Interface de R pour lês Analyses Multidimensionnelles de Textes et de Questionnaires) software. For the analysis of the textual corpus, descending hierarchical classification (DHC) was chosen.

### Ethical Aspects

This study complied with the ethical guidelines established by Resolution 466/2012 of the National Health Council^(19)^ and was submitted to and approved by the Research Ethics Committee of the Federal University of Piauí (CEP/UFPI), with opinion no. 5,218,237.

The anonymity of the participants was preserved, and the accounts from the statements were identified by the code A. (for adolescent) followed by an identity number for the group (e.g., A1, A2, A3, and so on).

## RESULTS

Six FGs (one FG/school) were conducted, with an average duration of 51 minutes, with the participation of 45 adolescents, with an average age of 15.88 (±1.43) years, most of whom were female (51.2%), self-declared brown-skinned (48%), Catholic (57.1%), single, without a steady partner (53.7%), and living with their parents (58.6%).

**Figure 1:**
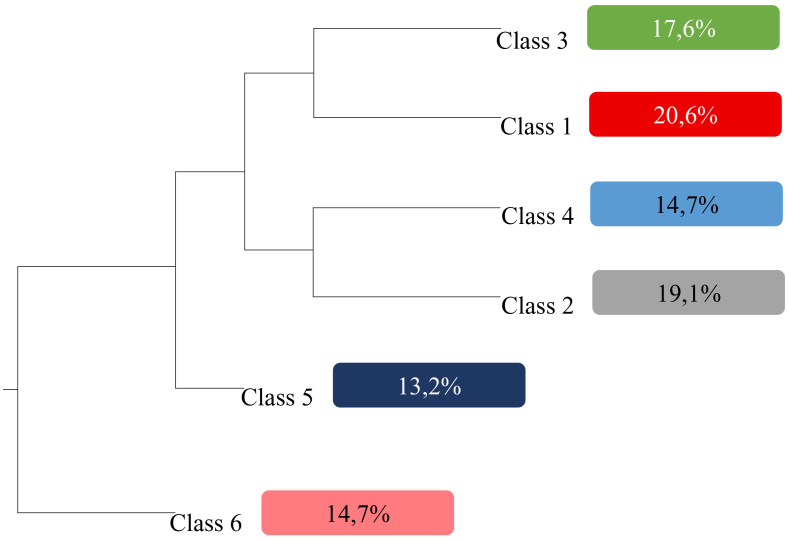
Dendrogram of the classes obtained from the textual corpus.

The analysis of the textual corpus generated 47 text units, 87 text segments, 556 distinct forms, and 2,655 word occurrences. According to the descending hierarchical analysis, the material was organized into six semantic classes, as shown in Figure 1.

The classes were then divided into three branches (1, 2, 3) of the textual corpus, and the semantic proximities between them were identified (Figure 1). These were regrouped, generating three categories: perception of risk and susceptibility (classes 1 and 3); severity and seriousness of COVID-19 (classes 2 and 4); perception of prevention and use of preventive measures against coronavirus infection (classes 5 and 6) (Chart 1).

**Chart 1:** Demonstration of classes and categories – Picos, PI, Brazil, 2023.

Class	Categories
Class 3	Perception of risk and susceptibility to COVID-19
Class 1	
Class 4	Severity and gravity of COVID-19
Class 2	
Class 5	Prevention and protective measures against coronavirus contamination
Class 6	

Source: Authors (2023).

### Category 1 – Perception of Risk and Susceptibility to COVID-19

Classes 01 and 03 comprised 38.2% of the segments, representing more than a third of the adolescents’ statements, who expressed anxiety, feelings, fears, and ideas about risk perception, vulnerability, and susceptibility to COVID-19, expressed through words such as threat, cause, death, getting sick, and transmitting.

*In this case [COVID-19] is not [just a risk] for adolescents, it is for everyone, because it causes deaths. It is like a threat, visiting the elderly, not shaking hands [...] (A1).*

*Adolescents are [afraid] of dying, afraid of getting sick[...] taking precautions [is a way] of not passing it on to other people (A4).*

*[...] [there is a risk of Covid-19] being transmitted to other people such as children and the elderly (A28).*

*[...] at first I thought it wouldn’t be a threat to me, and people said that it was mostly the elderly and who was sick , but then many cases started to appear among young people and I became concerned (A18).*

*[...] I distanced myself and wore a mask because there are elderly people at home. If I got it, it would be more risky for them (A2).*

*We made video calls to friends (A7).*

### Category 2 – Severity and Gravity of COVID-19

Classes 02 and 04 accounted for 33.8% of the textual segments with semantic similarity regarding the recognition of morbidity and mortality and severity related to infection with the new coronavirus. The statements showed that the moment they experienced was difficult, due to the need to stay at home and the intensification of hygiene measures. Thus, the most frequent words in the classes were: home, alcohol, wearing a mask, isolation, and fear.

The adolescents’ reports highlight their understanding of the gravity and severity of COVID-19, exemplified by their fear of dying, concern for the health of family members, especially the most vulnerable, and the need to adopt protective measures such as wearing masks and improving hygiene habits.

*[...] it was a time we lost, and at that time, we couldn’t go out, have contact with other people, [because] the disease is a threat to adolescents. [Being] with friends was no longer a frequent reality, and with [online] classes, we stopped socializing, going to places where there were crowds because of fear (A13).*

*Those who survive are left with sequelae; the threat is related to illness and death [...] I sometimes wore a mask, [out of] fear of dying (A3).*

*[referring to COVID-19]. Because it caused many deaths, I was afraid of losing my sense of taste and smell, [....] afraid for my sister who was pregnant and had my niece during the pandemic, she was very small (A12).*

*Because they were suffering until they died [referring to people with COVID-19], I wore a mask, improved my hygiene, stayed at home, and got vaccinated. In terms of feeling alone, I think it doesn’t matter [...] (A19).*

*When I got Covid, I cried almost all the time, afraid that the worst would happen (A22).*

*Because nowadays there is a vaccine and there is practically no more serious virus, (fear) of transmitting it to my parents, I wore a mask and used hand sanitizer [...] (A 24).*

*I wasn’t afraid, nor did I catch it, but I wore a mask and still use hand sanitizer today, I kept my distance at times, I avoided crowds and avoided enclosed spaces (A. 46).*

The perception of the gravity and severity of the moment led adolescents to face a new reality in the way they live and relate to each other, as well as to adapt to the context of crisis.

*[referring to COVID-19]. I didn’t think it would affect young people because they have high immunity, until it reached my home and ended up taking my grandmother. It was a time when there was no vaccine, nothing (A14).*

*Because everyone was socializing, it was Carnival season, and then everything had to stop (A11).*

*[...] making friends, then most people became shy because we got very used to being at home (A13).*

*Covid-19 affected our entire generation and completely changed the way we take care of ourselves. At first, it took everyone by surprise, no one was expecting it, and suddenly many people started dying and the symptoms worsened (A. 17).*

### Category 3 – Prevention and Protective Measures against Coronavirus Contamination

This category of analysis covers classes 05 and 06 and represents 27.9% of the textual corpus. Among the most frequent words in these classes are: mask, alcohol, gel, avoid, health, immunity, hand, hygiene, crowding, distancing, social isolation.

The participants’ statements illustrate the strategies for preventing coronavirus infection that were adopted by adolescents during the pandemic to break the chain of transmission, especially social distancing.

*Hygiene, especially when coming in from the street, vitamin C helps with immunity, wearing a mask, and washing your hands (A. 36).*

*[...] wearing a mask, washing your hands because our hands are touching various places [...] (A. 37).*

*There are ways to prevent it, such as staying at home, because it is a deadly disease. At school, [referring to social events] parties, I wore a mask and still use hand sanitizer today, I kept my distance at times; I avoided crowds and avoided enclosed spaces (A.43, A.47).*

*Avoid leaving home because adolescents have greater immunity, so if they take precautions, they will not pass it on to other people, avoid contact with the elderly (A. 36).*

*It was difficult to stay away from the people we love, from our friends (A. 34).*

Adolescents indicated immunity and access to health services as preventive measures related to the control, prevention, and reduction of coronavirus transmission. Most said they did not need health care during the period, but when they did, they were able to get it.

*[...] I received health care. I needed to have tests done and was able to, but the priority was Covid (A. 38).*

## DISCUSSION

The findings of this study show that adolescents developed an expanded perception of the Covid-19 pandemic, organized into three central analytical axes: perception of risk and susceptibility, severity and seriousness of the disease, and perception of prevention with the adoption of preventive measures. These findings reinforce the evidence from the literature which points out that, although adolescents are often described as a group with a lower perception of individual risk, in contexts of health crises this population demonstrates an ability to understand threats to health and to adjust behaviors in the face of collective risks^(20,21)^.

The perception of risk and susceptibility identified in classes 1 and 3 reveal that adolescents recognized the possibility of infection both for themselves and for those close to them, especially family members. This recognition is a central component of the Health Belief Model, according to which the perception of susceptibility directly influences the adoption of protective behaviors^(13)^. Studies conducted during the pandemic indicate that adolescents who perceive greater personal and social risk tend to adhere more consistently to health recommendations, such as social distancing and the use of masks, corroborating the findings of this study^(22,23)^.

The Health Belief Model has proven important for understanding how adolescents perceive and respond to an emerging crisis, such as the COVID-19 pandemic, suggesting that the perception of vulnerability and severity of the disease, combined with the belief in the effectiveness of preventive measures, influence individuals’ health behavior. In the case of adolescents, the perception of the severity of COVID-19 and the belief in the effectiveness of recommended practices to control the pandemic were decisive factors in the adoption of preventive behaviors during this period^(24,25)^.

In this sense, health care among adolescents must be reinforced, giving them the opportunity to understand the perception of risk, susceptibility, severity, and seriousness of behavior and/or exposure to health hazards for decision-making on preventive measures.

Regarding the gravity and severity of COVID-19, represented by classes 2 and 4, the statements show that adolescents understood the pandemic as a high-impact event capable of changing routines, social relationships, and life plans. This perception is in line with the construct of perceived severity of the health belief model, which refers to the assessment of the physical, social, and emotional consequences of a disease^(13)^. Although COVID-19 has a lower lethality rate among adolescents, studies indicate that the perception of severity was strongly associated with the fear of transmitting the virus to family members, especially the elderly, and the psychosocial repercussions of social isolation^(6,26)^.

The category related to the perception of prevention and use of preventive measures (classes 5 and 6) shows that adolescents assimilated the information about strategies to control the transmission of the coronavirus, namely social distancing, hand hygiene, and the use of masks being the most mentioned practices. These findings suggest the effectiveness of health communication actions developed in the country during the pandemic, in line with guidelines from the Ministry of Health and the World Health Organization^(27,28)^. Furthermore, the mention of immunity and access to health services as preventive measures demonstrate a broader understanding of health care, which involves both individual behaviors and the organization of health systems.

These results directly dialogue with public health and education policies in the Brazilian context, especially with the principles of the Unified Health System (SUS), such as universality, comprehensiveness, and equity, and with the guidelines of the National Health Promotion Policy (PNPS in the Portuguese acronym)), which emphasizes the protagonism of individuals and the strengthening of autonomy for care^(29)^. In addition, the articulation between health and education^(30)^, provided for in the Health at School Program (PSE in the Portuguese acronym), is fundamental for the development of educational actions aimed at adolescents, especially in health emergencies, in which schools and digital environments become strategic spaces for the dissemination of reliable information.

It is noteworthy that most adolescents reported not needing health care during the pandemic period, but those who sought assistance were able to access services. This data may reflect the perception of the effectiveness of the SUS, despite the difficulties imposed by the pandemic, and act as a protective factor by reinforcing confidence in the health system.

Overall, the results indicate that the COVID-19 pandemic acted as a catalyst for the development of health awareness among adolescents, putting them on alert and promoting understanding of individual and collective risk^(24,25)^. These findings reinforce the need to recognize adolescents as active subjects in public health policies, capable of understanding complex information and participating responsibly in prevention strategies. Thus, investing in educational actions based on theoretical models, such as the health beliefs model, integrated with SUS and educational sector policies, can strengthen health promotion and prepare this population to face future health crises.

It should be noted that the data presented in this study were based exclusively on self-reported responses from adolescents, which may be subject to social desirability bias, limited to a specific geographical and cultural context. However, the findings can help to understand how adolescents coped during this period, as well as the possible impacts on the health and development of this population, providing a basis for health actions aimed at mitigating these impacts.

## CONCLUSION

The study demonstrated how the Covid-19 pandemic affected the lives of adolescents, which was accompanied by distinct and complex perceptions of risk, susceptibility, and severity. In particular, the perception of risk and severity of the health crisis contributed to adolescents adopting self-protective behaviors for themselves and their families. Of particular note are the manifestations of feelings of fear, anxiety, isolation, and difficulties that may have consequences on the mental health and social interactions of adolescents in the post-pandemic period.

The findings reinforce the strategic role of nursing in mediating between information, risk perception, and the adoption of preventive behaviors among adolescents. In healthcare practice, PHC and PSE nurses can develop educational actions based on the health beliefs model, such as: conversation circles that explore the perception of susceptibility and severity of communicable diseases; brief counseling strategies for adolescents, incorporating examples from school and family life; use of accessible language and digital resources to reinforce the perceived benefits of preventive measures.

The study recommends that the health beliefs model helps adolescents to be ready to adopt preventive behaviors, as it is anchored in an understanding of personal risk and their environment, the susceptibility and severity of the disease, and/or exposure to health hazards, whether at the individual or collective level.

With regards to the school context, it suggests that topics such as the prevention of communicable diseases and critical thinking about health information be included across the curriculum, strengthening adolescents’ health literacy. The articulation between health and education can contribute to more consistent responses to future health emergencies.

Regarding public policies, the findings indicate that psychosocial determinants that influence adolescent behavior, such as beliefs, values, social norms, and access to health services, should be considered.

For future research, we recommend the development of longitudinal studies to monitor the maintenance of preventive behaviors over time, comparative analyses between different regions and socioeconomic realities, intervention studies conducted by nurses, evaluating educational strategies based on the health beliefs model, and studies that integrate qualitative and quantitative methods to deepen the understanding of adolescents’ beliefs and attitudes toward health crises.

## Data Availability

The entire dataset supporting the results of this study is available upon request to the corresponding author.
