# Supplementary material for: From the perceived risks to the preventative actions: analysis of adolescents’ behaviors facing Covid-19
Source: Rev Esc Enferm USP. 2026 Apr 17;60:e20250389. doi: 10.1590/1980-220X-REEUSP-2025-0389en (PMC13232737; doi:10.1590/1980-220X-REEUSP-2025-0389en)
Supplement: Translation

# Translation: Entre o risco percebido e a ação preventiva: análise do comportamento de adolescentes diante da Covid-19*

**DOI:** 10.1590/1980-220X-REEUSP-2025-0389pt

**Authors:** Jayne Ramos Araújo Moura, Mariana Ribeiro Silva, João Rafael da Silva Fonseca, Jaqueline Renata da Silva Brito, Roberta Meneses Oliveira, Ana Larissa Gomes Machado, Neiva Francenely Cunha Vieira

**Affiliations:** 1 Universidade Federal do Ceará, Departamento de Enfermagem, Fortaleza, CE, Brasil.; 2 Universidade Federal do Piauí, Departamento de Enfermagem, Picos, PI, Brasil.; 3 Universidade Federal do Piauí, Departamento de Enfermagem, Teresina, PI, Brasil.

**Keywords:** COVID-19, Coronavirus, Comportamento do Adolescente, Modelo de Crenças de Saúde, Isolamento Social.

## Abstract

**Objetivo:**

Compreender as respostas de adolescentes à pandemia de Covid-19, considerando suas crenças e atitudes diante da ameaça de contágio e transmissibilidade.

**Método:**

Estudo qualitativo, fundamentado no referencial teórico do Modelo de Crenças em Saúde, realizado em município do Nordeste brasileiro. Foram realizados seis grupos focais, com a participação de 45 adolescentes. As falas foram transcritas, organizadas em corpus textual e submetidas à análise de conteúdo no *software* IRAMUTEQ.

**Resultados:**

Emergiram três categorias temáticas: 1. Percepção de risco e suscetibilidade à Covid-19 – ameaça, morte; 2. Gravidade e severidade da Covid-19 – isolamento, medo; 3. Prevenção e medidas protetoras à contaminação pelo coronavírus – distanciamento social, higiene.

**Conclusão:**

O estudo demonstrou que a percepção de risco e gravidade da crise sanitária contribuiu para que os adolescentes adotassem comportamentos de autoproteção para si e sua família. De maneira que a saúde mental destes foi afetada pelos sentimentos de medo, ansiedade, refletidos nas interações sociais no período de isolamento e pós-pandêmico. Sugere-se que haja maior direcionamento de ações em saúde para este público, bem como estudos que identifiquem outros desfechos do período experienciado.

## INTRODUÇÃO

O período pandêmico da Covid-19 consistiu em um momento crucial para compreender como as pessoas respondem às situações de crise sanitária e conhecer os fatores que desencadeiam a prontidão para agir individualmente e coletivamente no controle e prevenção de agravos.

A população adolescente, mesmo não sendo o grupo mais vulnerável durante a pandemia, sofreu perdas familiares e foi submetida ao isolamento social que a impediu de conviver com seus pares nesse estágio de transição para a vida adulta. Estudos têm demonstrado os efeitos psicossociais que afetaram o crescimento e o desenvolvimento de adolescentes, os quais ainda permanecem no período pós-pandêmico^(1,2)^.

Paralelamente, ocorreram uma série de transformações nos recursos tecnológicos para aprendizagem que impactaram significativamente a vida cotidiana dos adolescentes, especialmente na manutenção das atividades escolares e na comunicação durante o período de isolamento social. Embora essas mudanças tenham possibilitado a continuidade do ensino por meio de plataformas digitais, a suspensão das aulas presenciais e a transição para o ensino remoto foram associadas a um aumento expressivo dos sintomas de depressão e ansiedade nesta população^(3,4)^.

Além das dificuldades do seguimento curricular de ensino, a ausência da rotina escolar presencial e a limitação das interações sociais amplificaram os sentimentos de solidão e isolamento, impactando negativamente na saúde mental dos adolescentes^(5)^. A restrição de atividades extracurriculares e a falta de espaços de convivência agravaram a vulnerabilidade emocional dos jovens, refletindo-se em uma redução da qualidade no processo da aprendizagem, produtividade e no aumento da prevalência de transtornos mentais^(6)^.

Apesar desses desafios, adolescentes demonstraram resiliência ao buscar formas alternativas para manter a socialização e o aprendizado por meio de ferramentas digitais, conectando- se com amigos e familiares e acessando informações confiáveis sobre a pandemia^(7,8)^.

Estudos sobre o comportamento de saúde dos adolescentes, de uma maneira geral, têm abordado distintas situações de riscos como gravidez não planejada, infecções sexualmente transmissíveis e uso abusivo de álcool e drogas. As evidências demonstraram que as relações interpessoais exerceram influências nas respostas dos adolescentes quanto a adoção de medidas preventivas, incluindo a relação com seus pares^(9,10,11)^.

Ainda sobre esse aspecto, a Pesquisa Nacional de Saúde Escolar tem reiterado as disparidades regionais diante dos agravos que atingem os adolescentes, o que enseja o fortalecimento de programas educativos mais acessíveis a esse público. A região Nordeste concentrou o menor percentual de mães que possuíam ensino superior, menores proporções de adolescentes fisicamente ativos, maior percentual de gravidez na adolescência, elevada proporção de padrão de alimentação menos saudável, quando comparada as demais regiões do país^(11)^.

Um dos aspectos importantes destacados neste estudo foi o fato de que a crise sanitária advinda da pandemia da Covid-19 atingiu todo o ciclo vital familiar e, ao mesmo tempo, suas condições de existência, expondo situações de enfrentamento, como perdas, lutos, restrições de ordem econômica e social. Os efeitos desta fase puderam trazer à luz elementos que conversam com outros segmentos do desenvolvimento dos adolescentes, como a convivência (ou ausência) no âmbito escolar e as interações com seus pares que conformam a sua personalidade e identidade na realidade social. Reforçando a ideia de que o processo educativo para o adolescente deve dialogar com fatores do seu entorno, vínculos afetivos, inovando soluções para o bem-estar coletivo.

Diante disso, o enfermeiro, inserido nos serviços e sistemas de saúde, em especial na Atenção Primária à Saúde (APS), atua como elo importante na cadeia de promoção e proteção à saúde dos adolescentes, em especial na articulação de ações envolvendo outros sistemas, como escolas, desempenhando ações que visem desde a assistência direta até a produção de conhecimentos que baseiem a prática clínica.

Este estudo, portanto, teve como objetivo compreender as respostas dos adolescentes à pandemia de Covid-19, considerando suas crenças e atitudes diante da ameaça de contágio e transmissibilidade. A pesquisa pode contribuir para identificar os fatores que influenciam os comportamentos de saúde dos adolescentes diante de crises emergenciais, fundamentando- se no modelo de crenças em saúde como referencial teórico.

## MÉTODO

### Tipo ou Desenho do Estudo

Estudo qualitativo, desenvolvido com base nos critérios do *Consolidated Criteria for Reporting Qualitative Research* (COREQ)^(12)^, fundamentado no referencial teórico do Modelo de Crenças em Saúde (MCS), que permite compreender como a percepção do risco de uma doença e a crença na eficácia das medidas preventivas influenciam diretamente os comportamentos individuais^(13)^.

O MCS foi utilizado para entender os motivos pelos quais adolescentes aderiram a medidas preventivas durante a pandemia, como o uso de máscaras e o distanciamento social, enquanto outros não. Ademais, fatores como autoeficácia e motivação interna são relevantes para a adoção de práticas de prevenção e enfrentamento do estresse associado ao isolamento social imposto pela crise sanitária, os quais podem ser mais bem compreendidos a partir do referencial.

### Local

O estudo foi realizado em um município situado no interior de um estado do Nordeste brasileiro, em região de semiárido, com população estimada de 78.002 habitantes, índice de desenvolvimento humano (IDH) de 0,698, e com severas desigualdades sociais^(14)^, no período de novembro de 2021 a novembro de 2023.

### População e Critérios de Seleção

A população do estudo foi composta por adolescentes de ambos os sexos, matriculados nas turmas de 8º e 9º do ensino fundamental e ensino médio de escolas da rede pública de ensino, localizadas na zona urbana, na faixa etária entre 13 e 18 anos.

Para seleção dos participantes foram considerados os seguintes critérios de inclusão: estar matriculado e comparecer à escola nos dias da realização da coleta de dados; e, exclusão: apresentar limitação cognitiva que impeça a participação do estudo, deficiência visual e/ou auditiva que exigiam a necessidade de apoio pedagógico especial para realização das tarefas escolares.

### Definição da Amostra

Os adolescentes foram selecionados por meio de amostragem intencional e o quantitativo de participantes foi definido quando alcançada a redundância informacional, ou seja, por saturação teórica^(15,16)^.

### Coleta de Dados

Para a coleta de dados, adotou-se a técnica de Grupo Focal (GF)^(17)^. Os GFs foram mediados a partir de um roteiro de perguntas abertas que suscitaram a discussão, com foco para as estratégias de enfrentamento diante as adversidades ocasionadas pela pandemia da Covid-19, organizado em quatro momentos: (a) apresentação dos participantes; (b) dinâmica quebra-gelo; (c) perguntas disparadoras com foco na compreensão das percepções e experiências dos adolescentes na pandemia do Covid-19 (d) finalização.

As perguntas disparadoras foram as seguintes: A Covid-19 é uma ameaça ao adolescente? Por que o Covid-19 é uma ameaça para a saúde dos adolescentes? Na opinião de vocês em que locais a Covid-19 é maior ameaça e por quê? Pensando em todo o período da pandemia, quais os principais medos que vocês tiveram em relação a doença da Covid-19? Por que acreditavam que poderiam ter sintomas graves? Quais estratégias vocês adotaram para se prevenir do coronavírus? E, na opinião de vocês, como o adolescente pode auxiliar no combate ao coronavírus? Qual ou quais eram as principais dificuldades no enfrentamento do coronavírus? Por quê? Como foi para vocês a época da Covid-19 com relação a escola, amigos, lazer etc.? Como se sentiram? Quais as consequências desse período para vocês? O que o que te motiva a tomar medidas preventivas contra o coronavírus? Quando precisaram, tiveram acesso à atendimento em saúde e/ou medicamentos, como foi essa experiência?

Os GFs foram compostos por grupos pequenos/homogêneos, entre 6 e 10 participantes, com duração aproximada de uma hora, gravados com equipamento específico.

### Análise e Tratamento dos Dados

Os dados gerados foram organizados trilhando as seguintes etapas: transcrição na íntegra, elaboração do inventário, que consiste em isolar os elementos presentes nas falas e expressões dos adolescentes, e geração do corpus textual.

Nessa fase de geração do corpus textual, utilizou-se análise de Conteúdo Reflexiva, um método trans teórico e flexível orientado para a descrição e redução de dados qualitativos manifestos. Trata-se de uma técnica usada para identificar padrões nos significados superficiais explícitos dos dados qualitativos por meio da aplicação de uma estrutura hierárquica de estratos analíticos quantificáveis chamados códigos, subcategorias e categorias. Cada estrato existe em um continuum de abstração, com os códigos sendo os mais próximos dos dados originais e as categorias sendo as mais abstratas. Durante cada etapa do processo, a reflexividade é considerada um recurso analítico valioso que é crucial para garantir uma descrição adequada dos dados. A técnica destina-se a ser usada como um método de análise de dados, e não uma metodologia; portanto, pode ser integrada com várias abordagens metodológicas e epistemológicas^(18)^.

Após essa organização, o corpus textual foi submetido à análise no software IRAMUTEQ (Interface de R pour lês Analyses Multidimensionnelles de Textes et de Questionnaires). Para a análise do corpus textual, foi escolhida a classificação hierárquica descendente (CHD).

### Aspectos Éticos

Este estudo respeitou as diretrizes éticas estabelecidas pela Resolução 466/2012, do Conselho Nacional de Saúde^(19)^, e foi submetido e aprovado pelo Comitê de Ética em Pesquisa Universidade Federal do Piauí (CEP/UFPI), com o parecer nº. 5.218.237.

O anonimato dos participantes foi preservado, e os trechos das falas foram identificados pelo código A. (de adolescente) seguido de uma numeração identitária do grupo (Ex.: A1, A2, A3, e assim por diante).

## RESULTADOS

Foram realizados seis GFs (um GF/escola), com duração média de 51 minutos, com a participação de 45 adoles­centes, com idade média de 15,88 (±1,43) anos, a maioria do sexo feminino (51,2%), cor autodeclarada parda (48%), religião católica (57,1%), solteira, sem parceiro fixo (53,7%), morando com os pais (58,6%).

A análise do *corpus* textual gerou 47 unidades de texto, 87 segmentos de texto, 556 formas distintas e 2.655 ocorrências de palavras. De acordo com a análise hierárquica descendente, o material foi organizado em seis classes semânticas, como observado na Figura 1.

**Figura 1:** Dendograma das classes obtidas a partir do corpus textual.

As classes foram, então, divididas em três ramificações (1, 2, 3) do *corpus* textual, e identificadas as proximidades semânticas entre si (Figura 1). Estas foram reagrupadas, gerando três categorias: percepção de risco e susceptibilidade (classes 1 e 3); gravidade e severidade da Covid-19 (classes 2 e 4), percepção de prevenção e uso de medidas preventivas à infecção pelo coronavírus (classes 5 e 6) (Quadro 1).

**Quadro 1:** Demonstrativo das classes e categorias – Picos, PI Brasil, 2023.

Classe	Categorias
Classe 3	Percepção de risco e suscetibilidade à Covid-19
Classe 1	
Classe 4	Gravidade e severidade da Covid-19
Classe 2	
Classe 5	Prevenção e medidas protetoras à contaminação pelo coronavírus
Classe 6	

Fonte: Autores (2023).

### Categoria 1 – Percepção de Risco e Susceptibilidade à Covid-19

As classes 01 e 03 compreenderam 38,2% dos segmentos, o que representa mais de um terço das falas dos adolescentes, que manifestaram ansiedade, medos e ideia de percepção de risco, vulnerabilidade e susceptibilidade à Covid-19, expressas por meio de palavras como ameaça, causa, morte, adoecer, transmitir.

*No caso [a covid-19] não [é só um risco] para o adolescente, é para todos, porque causa mortes. É tipo uma ameaça, de visitar idoso, não apertar as mãos [...] (A1).*

*O adolescente tem [medo] de morrer, medo de pegar a doença [...] se prevenindo [é uma forma de] não passar para as outras pessoas (A4).*

*[...] [existe risco da Covid-19] transmitir para outras pessoas como crianças e idosos (A28).*

*[...] no início eu achei que não iria ser uma ameaça pra mim, e as pessoas falavam que quem pegava mais eram os idosos, quem era doente, mas aí depois começou a surgir muitos casos de jovens e aí fiquei preocupada (A18).*

*[...] eu fiz distanciamento, usei máscara, porque lá em casa tem idosos. Se eu pegasse, ia ser mais arriscado para eles (A2).*

*A gente ficava fazendo chamada de vídeo com os amigos (A7).*

### Categoria 2 – Gravidade e Severidade da Covid-19

As classes 02 e 04 reuniram 33,8% dos segmentos textuais com similaridade semântica quanto ao reconhecimento da morbimortalidade e gravidade relacionado à infecção pelo novo coronavírus. As falas demonstraram que o momento vivenciado por eles foi difícil, pela necessidade de ficar em casa e intensificação da adoção de medidas de higiene. Desse modo, as palavras mais frequentes nas classes foram: casa, álcool, usar máscara, isolamento e medo.

Os relatos dos adolescentes destacam a compreensão da gravidade e severidade da Covid-19, exemplificados pelo medo de morrer, a preocupação com a saúde dos familiares, especialmente de pessoas mais vulneráveis, e a necessidade de adotar medidas de proteção como o uso de máscaras e a melhoria dos hábitos de higiene.

*[...] foi um tempo que a gente perdeu e, nessa época, não podia sair, ter contato com outras pessoas, [pois] a doença é uma ameaça ao adolescente. [Estar] com os amigos deixou de ser uma realidade frequente, e com as aulas [online] a gente parou de conviver, de ir a lugares que tinha aglomerações por conta do medo (A13).*

*Quem sobrevive fica com sequelas, a ameaça está relacionada a doença e morte [...] eu às vezes usei máscara, [com] medo de morrer (A3).*

*[referindo-se Covid-19]. Porque causou muitas mortes, eu tinha medo de perder o paladar e o cheiro, [....] medo pela irmã que estava grávida e teve a minha sobrinha na pandemia, ela era muito pequena (A12).*

*Porque ficavam sofrendo até morrer [referindo-se as pessoas com Covid-19], usei máscara, melhorei os aspectos de higiene e fiquei em casa, tomei vacina. Em questão de se sentir sozinho acho que tanto faz [...] (A19).*

*Quando eu peguei covid eu chorava quase o tempo todo com medo de acontecer o pior (A22).*

*Porque hoje em dia tem a vacina e praticamente não tem mais o vírus grave, (medo) de transmitir para meus pais, usei máscara e álcool em gel [...](A 24).*

*Eu não tive medo, nem peguei, mas usei máscara e uso até hoje álcool em gel, mantive distanciamento as vezes, eu evitei aglomeração e evitei lugares fechados (A. 46).*

A percepção da gravidade e severidade do momento vivido provocaram nos adolescentes a assimilação de uma nova realidade, na forma de viver e se relacionar, como também de adaptação ao contexto de crise.

*[referindo-se Covid-19]. Eu não achava que iria atingir o jovem, porque tem a imunidade alta, até o ponto de atingir a minha casa e acabou levando a minha avó, era uma época que não tinha vacina, nada (A14).*

*Porque estava todo mundo se socializando era época do carnaval e aí teve que parar tudo (A11).*

*[...] de fazer amizades, depois a maioria ficou tímido porque a gente ficou muito acostumado a estar em casa (A13).*

*A Covid-19 afetou toda a nossa geração e mudou totalmente o jeito da gente se cuidar, no início pegou todo mundo de surpresa, ninguém estava esperando e de repente muita gente começou a morrer e os sintomas se agravarem (A. 17).*

### Categoria 3 – Prevenção e Medidas Protetoras à Contaminação pelo Coronavírus

Esta categoria de análise contempla as classes 05 e 06, e representa 27,9% do corpus textual. Dentre as palavras mais frequentes das classes estão: máscara, álcool, gel, evitar, saúde, imunidade, mão, higiene, aglomeração, distanciamento, isolamento social.

As falas dos participantes ilustram as estratégias de prevenção da infecção pelo coronavírus que foram adotadas pelos adolescentes durante a pandemia para quebrar a cadeia de transmissão, em especial o distanciamento social.

*Higiene, principalmente, quando vier da rua, a vitamina c ajuda na imunidade, usar máscara e levar as mãos (A. 36).*

*[...] usar máscara, lavar as mãos porque a nossa mão está tocando em vários lugares [...] (A. 37).*

*Tem os meios de se prevenir, como ficar em casa, porque é uma doença que mata. Nas escolas, [referindo-se a eventos sociais] festas, usei máscara e uso até hoje álcool em gel, mantive distanciamento às vezes; eu evitei aglomeração e evitei lugares fechados (A.43, A.47).*

*Evitar sair de casa porque o adolescente tem uma maior imunidade, então se ele se prevenir não vai passar para outras pessoas, evitar ter contato com os idosos (A. 36).*

*Foi difícil ficar longe das pessoas que a gente gosta, dos amigos (A. 34).*

Os adolescentes indicaram a imunidade e o acesso aos serviços de saúde como medidas preventivas relacionadas ao controle, prevenção e redução da transmissão do coronavírus. A maioria afirmou não ter precisado de atendimento em saúde durante o período, porém, quando houve necessidade, conseguiram atendimento.

*[...] tive atendimento de saúde. Eu precisei fazer exames e consegui, mas a prioridade era o Covid (A. 38.)*

## DISCUSSÃO

Os achados deste estudo evidenciam que os adolescentes desenvolveram uma percepção ampliada acerca da pandemia da covid-19, organizada em três eixos analíticos centrais: percepção de risco e suscetibilidade, gravidade e severidade da doença, e percepção da prevenção com adoção de medidas preventivas. Esses resultados reforçam evidências da literatura as quais apontam que, embora adolescentes sejam frequentemente descritos como um grupo com menor percepção de risco individual, em contextos de crise sanitária essa população demonstra capacidade de compreender ameaças à saúde e de ajustar comportamentos frente a riscos coletivos^(20,21)^.

A percepção de risco e suscetibilidade, identificada nas classes 1 e 3, revela que os adolescentes reconheceram a possibilidade de infecção tanto para si quanto para pessoas próximas, especialmente familiares. Tal reconhecimento é um componente central do Modelo de Crenças em Saúde, segundo o qual a percepção de suscetibilidade influencia diretamente a adoção de comportamentos protetivos^(13)^. Estudos realizados durante a pandemia indicam que adolescentes que percebem maior risco pessoal e social tendem a aderir mais consistentemente às recomendações sanitárias, como o distanciamento social e o uso de máscaras, corroborando os achados deste estudo^(22,23)^.

O modelo de crenças em saúde mostrou-se importante para entender como os adolescentes percebem e respondem a uma crise emergente, como a pandemia da Covid-19, sugerindo que a percepção de vulnerabilidade e gravidade da doença, aliada à crença na eficácia das medidas preventivas, influenciam no comportamento de saúde dos indivíduos. No caso dos adolescentes, a percepção da gravidade da Covid-19 e a crença na eficácia das práticas recomendadas para conter a pandemia foram determinantes para a adoção de comportamentos preventivos durante este período^(24,25)^.

Nesse sentido, se deve reforçar o cuidado da saúde entre os adolescentes, dando-lhes oportunidade de compreensão da percepção de risco, susceptibilidade, gravidade e severidade de comportamento e/ou exposição de agravo à saúde para tomada de decisão de medidas preventivas.

No tocante à gravidade e severidade da Covid-19, representadas pelas classes 2 e 4, as falas demonstram que os adolescentes compreenderam a pandemia como um evento de grande impacto, capaz de modificar rotinas, relações sociais e projetos de vida. Essa percepção está alinhada ao constructo de severidade percebida do modelo de crenças em saúde, que se refere à avaliação das consequências físicas, sociais e emocionais de uma doença^(13)^. Ainda que a Covid-19 apresente menor letalidade entre adolescentes, estudos indicam que a percepção da gravidade esteve fortemente associada ao medo de transmitir o vírus a familiares, especialmente as pessoas idosas, e às repercussões psicossociais do isolamento social^(6,26)^.

A categoria relacionada à percepção de prevenção e uso de medidas preventivas (classes 5 e 6) evidencia que os adolescentes assimilaram informações sobre estratégias de controle da transmissão do coronavírus, sendo o distanciamento social, a higiene das mãos e o uso de máscaras as práticas mais mencionadas. Esses achados sugerem a efetividade das ações de comunicação em saúde desenvolvidas no país durante a pandemia, em consonância com diretrizes do Ministério da Saúde e da Organização Mundial da Saúde^(27,28)^. Ademais, a menção à imunidade e ao acesso aos serviços de saúde como medidas preventivas demonstra uma compreensão ampliada do cuidado em saúde, que envolve tanto comportamentos individuais quanto a organização dos sistemas de saúde.

No contexto brasileiro, tais resultados dialogam diretamente com as políticas públicas de saúde e educação, especialmente com os princípios do Sistema Único de Saúde (SUS), como a universalidade, integralidade e equidade, e com as diretrizes da Política Nacional de Promoção da Saúde (PNPS), que enfatiza o protagonismo dos sujeitos e o fortalecimento da autonomia para o cuidado^(29)^. Além disso, a articulação entre saúde e educação^(30)^, prevista no Programa Saúde na Escola (PSE), mostra-se fundamental para o desenvolvimento de ações educativas voltadas aos adolescentes, sobretudo em situações de emergência sanitária, nas quais a escola e os ambientes digitais tornam-se espaços estratégicos para a disseminação de informações confiáveis.

Destaca-se que a maioria dos adolescentes relatou não ter necessitado de atendimento em saúde durante o período pandêmico, mas aqueles que buscaram assistência conseguiram acesso aos serviços. Esse dado reflete a resolutividade do SUS, apesar das dificuldades impostas pela pandemia, e atuar como fator de proteção ao reforçar a confiança no sistema de saúde.

De modo geral, os resultados indicam que a pandemia da Covid-19 funcionou como um evento catalisador para o desenvolvimento da consciência em saúde entre adolescentes, ao colocá-los em estado de alerta e favorecer a compreensão do risco individual e coletivo^(24,25)^. Esses achados reforçam a necessidade de reconhecer os adolescentes como sujeitos ativos nas políticas públicas de saúde, capazes de compreender informações complexas e de participar de forma responsável nas estratégias de prevenção. Assim, investir em ações educativas baseadas em modelos teóricos, como o modelo de crenças em saúde, integradas às políticas do SUS e do setor educacional, pode fortalecer a promoção da saúde e a preparação dessa população para o enfrentamento de futuras crises sanitárias.

Convém pontuar que os dados apresentados neste estudo foram baseados exclusivamente em respostas autorrelatadas de adolescentes que podem estar sujeitas a viés de desejabilidade social, limitados a um contexto geográfico e cultural específico. No entanto, os achados podem auxiliar no entendimento de como os adolescentes enfrentaram esse período, bem como quais os possíveis impactos para a saúde e desenvolvimento deste público, embasando ações em saúde direcionadas para mitigação destes impactos.

## CONCLUSÃO

O estudo demonstrou como a pandemia da Covid-19 afetou a vida dos adolescentes, que foi acompanhada de percepções de risco, susceptibilidade e gravidade distintas e complexas. Em especial, a percepção de risco e gravidade da crise sanitária contribuiu para que os adolescentes adotassem comportamentos de autoproteção tanto para si, quanto para sua família. Ressaltando-se as manifestações de sentimentos de medo, ansiedade, isolamento e dificuldades que podem ter repercussões na saúde mental e nas interações sociais dos adolescentes no período pós-pandemia.

Os achados reforçam o papel estratégico da enfermagem na mediação entre informação, percepção de risco e adoção de comportamentos preventivos entre adolescentes. Na prática assistencial, enfermeiros da APS e do PSE podem desenvolver ações educativas baseadas no modelo de crenças em saúde, como: rodas de conversa que explorem a percepção de susceptibilidade e severidade de doenças transmissíveis; estratégias de aconselhamento breve para adolescentes, incorporando exemplos do cotidiano escolar e familiar; uso de linguagem acessível e recursos digitais para reforçar benefícios percebidos das medidas preventivas.

O estudo recomenda que o modelo de crenças em saúde auxilia na prontidão dos adolescentes para adotar comportamentos de prevenção, por estar ancorado em uma compreensão do risco pessoal e do seu entorno, da susceptibilidade e gravidade da doença e/ou exposição do agravo a saúde quer seja no nível individual ou coletivo.

No contexto escolar, sugere-se a inclusão transversal de temas como prevenção de agravos transmissíveis e pensamento crítico sobre informações em saúde, fortalecendo o letramento em saúde dos adolescentes. A articulação entre saúde e educação pode contribuir para respostas mais consistentes a futuras emergências sanitárias.

Quanto às políticas públicas, os achados indicam que devem ser considerados os determinantes psicossociais que influenciam o comportamento dos adolescentes, como crenças, valores, normas sociais e acesso aos serviços de saúde.

Para investigações futuras recomenda-se o desenvolvimento de estudos longitudinais que acompanhem a manutenção de comportamentos preventivos ao longo do tempo, análises comparativas entre diferentes regiões e realidades socioeconômicas, pesquisas de intervenção conduzidas pela enfermagem, avaliando estratégias educativas baseadas no modelo de crenças em saúde e estudos que integrem métodos qualitativos e quantitativos para aprofundar a compreensão das crenças e atitudes dos adolescentes frente a crises sanitárias.

## Data Availability

Todo o conjunto de dados que dá suporte aos resultados deste estudo está disponível mediante solicitação ao autor correspondente.

# Translation: Entre el riesgo percibido y la acción preventiva: análisis del comportamiento de los adolescentes ante la COVID-19*

**DOI:** 10.1590/1980-220X-REEUSP-2025-0389es

**Authors:** Jayne Ramos Araújo Moura, Mariana Ribeiro Silva, João Rafael da Silva Fonseca, Jaqueline Renata da Silva Brito, Roberta Meneses Oliveira, Ana Larissa Gomes Machado, Neiva Francenely Cunha Vieira

**Affiliations:** 1 Universidade Federal do Ceará, Departamento de Enfermagem, Fortaleza, CE, Brasil.; 2 Universidade Federal do Piauí, Departamento de Enfermagem, Picos, PI, Brasil.; 3 Universidade Federal do Piauí, Departamento de Enfermagem, Teresina, PI, Brasil.

**Keywords:** COVID-19, Coronavirus, Conducta del Adolescente, Modelo de Creencias sobre la Salud, Aislamiento Social.

## Abstract

**Objetivo:**

Comprender las respuestas de los adolescentes a la pandemia de COVID-19, teniendo en cuenta sus creencias y actitudes ante la amenaza de contagio y transmisibilidad.

**Método:**

Estudio cualitativo, basado en el marco teórico del Modelo de Creencias en Salud, realizado en un municipio del noreste de Brasil. Se llevaron a cabo seis grupos focales, con la participación de 45 adolescentes. Las declaraciones fueron transcritas, organizadas en un corpus textual y sometidas a análisis de contenido en el software IRAMUTEQ.

**Resultados:**

Surgieron tres categorías temáticas: 1. Percepción del riesgo y la susceptibilidad a Covid-19: amenaza, muerte; 2. Gravedad y severidad la COVID-19: aislamiento, miedo; 3. Prevención y medidas de protección contra la contaminación por coronavirus: distanciamiento social, higiene.

**Conclusión:**

El estudio demostró que la percepción del riesgo y la gravedad de la crisis sanitaria contribuyó a que los adolescentes adoptaran comportamientos de autoprotección para ellos y sus familias. De manera que su salud mental se vio afectada por sentimientos de miedo y ansiedad, reflejados en las interacciones sociales durante el periodo de aislamiento y pospandémico. Se sugiere que se orienten más las acciones en salud hacia este público, así como estudios que identifiquen otros resultados del período experimentado.

## INTRODUCCIÓN

El periodo pandémico de la COVID-19 ha sido un momento crucial para comprender cómo responden las personas a las situaciones de crisis sanitaria y conocer los factores que desencadenan la disposición a actuar de forma individual y colectiva en el control y la prevención de enfermedades.

La población adolescente, aunque no fue el grupo más vulnerable durante la pandemia, sufrió pérdidas familiares y se vio sometida a un aislamiento social que le impidió convivir con sus pares en esta etapa de transición a la vida adulta. Diversos estudios han demostrado los efectos psicosociales que afectaron el crecimiento y el desarrollo de los adolescentes, los cuales aún persisten en el periodo pospandémico^(1,2)^.

Paralelamente, se han producido una serie de transformaciones tecnológicas que han tenido un impacto significativo en la vida cotidiana de los adolescentes, especialmente en el mantenimiento de las actividades escolares y la comunicación durante el periodo de aislamiento social. Aunque estos cambios han permitido la continuidad de la enseñanza a través de plataformas digitales, la suspensión de las clases presenciales y la transición a la enseñanza a distancia se han asociado a un aumento significativo de los síntomas de depresión y ansiedad en esta población^(3,4)^.

Además de las dificultades para seguir el plan de estudios, la ausencia de la rutina escolar presencial y la limitación de las interacciones sociales amplificaron los sentimientos de soledad y aislamiento, lo que repercutió negativamente en la salud mental de los adolescentes^(5)^. La restricción de las actividades extracurriculares y la falta de espacios de convivencia agravaron la vulnerabilidad emocional de los jóvenes, lo que se reflejó en una reducción de la calidad del proceso de aprendizaje y la productividad, y en un aumento de la prevalencia de trastornos mentales^(6)^.

A pesar de estos desafíos, los adolescentes han demostrado resiliencia al buscar formas alternativas de mantener la socialización y el aprendizaje a través de herramientas digitales, conectándose con amigos y familiares y accediendo a información confiable sobre la pandemia^(7,8)^.

Los estudios sobre el comportamiento de salud de los adolescentes, en general, han abordado diferentes situaciones de riesgo, como el embarazo no planeado, las infecciones de transmisión sexual y el abuso de alcohol y drogas. Las evidencias han demostrado que las relaciones interpersonales han influido en las respuestas de los adolescentes en cuanto a la adopción de medidas preventivas, incluida la relación con sus pares^(9,10,11)^.

En relación con este aspecto, la Encuesta Nacional de Salud Escolar ha reiterado las disparidades regionales ante los problemas que afectan a los adolescentes, lo que requiere el fortalecimiento de programas educativos más accesibles para este público.

La región nordeste concentró el menor porcentaje de madres con educación superior, menores proporciones de adolescentes físicamente activos, mayor porcentaje de embarazos en la adolescencia y una elevada proporción de patrones alimenticios menos saludables, en comparación con las demás regiones del país^(11)^.

Uno de los aspectos importantes destacados en este estudio fue el hecho de que la crisis sanitaria derivada de la pandemia de COVID-19 afectó a todo el ciclo vital familiar y, al mismo tiempo, a sus condiciones de existencia, exponiéndolos a situaciones difíciles, como pérdidas, duelos y restricciones económicas y sociales. Los efectos de esta fase pudieron sacar a la luz elementos que dialogan con otros segmentos del desarrollo de los adolescentes, como la convivencia (o ausencia de ella) en el ámbito escolar y las interacciones con sus pares que conforman su personalidad e identidad en la realidad social. Reforzando la idea de que el proceso educativo del adolescente debe dialogar con factores de su entorno, vínculos afectivos, innovando soluciones para el bienestar colectivo.

Ante esto, los enfermeros, integrados en los servicios y sistemas de salud, especialmente en la Atención Primaria de Salud (APS), actúan como un eslabón importante en la cadena de promoción y protección de la salud de los adolescentes, especialmente en la articulación de acciones que involucran a otros sistemas, como las escuelas, realizando acciones que van desde la asistencia directa hasta la producción de conocimientos que fundamentan la práctica clínica.

Por lo tanto, este estudio tuvo como objetivo comprender las respuestas de los adolescentes a la pandemia de COVID-19, considerando sus creencias y actitudes ante la amenaza de contagio y transmisibilidad. La investigación puede contribuir a identificar los factores que influyen en los comportamientos de salud de los adolescentes ante crisis de emergencia, basándose en el modelo de creencias en salud como referencia teórica.

## MÉTODO

### Tipo o Diseño del Estudio

Estudio cualitativo, desarrollado según los criterios del *Consolidated Criteria for Reporting Qualitative Research* (COREQ)^(12)^, basado en el marco teórico del Modelo de Creencias en Salud (MCS), que permite comprender cómo la percepción del riesgo de una enfermedad y la creencia en la eficacia de las medidas preventivas influyen directamente en los comportamientos individuales^>(13)^.

El MCS se utilizó para comprender las razones por las que los adolescentes se adhirieron a las medidas preventivas durante la pandemia, como el uso de cubrebocas y el distanciamiento social, mientras que otros no lo hicieron. Además, factores como la autoeficacia y la motivación interna son relevantes para la adopción de prácticas de prevención y afrontamiento del estrés asociado al aislamiento social impuesto por la crisis sanitaria, que pueden comprenderse mejor a partir del marco teórico.

### Lugar

El estudio se llevó a cabo en un municipio de un estado del nordeste de Brasil, en una región semiárida, con una población estimada de 78 002 habitantes, un índice de desarrollo humano (IDH) de 0,698 y graves desigualdades sociales^(14)^, en el periodo comprendido entre noviembre de 2021 y noviembre de 2023.

### Población y Criterios de Selección

La población del estudio estuvo compuesta por adolescentes de ambos sexos, matriculados en los cursos de 8.º y 9.º de la enseñanza básica y secundaria de escuelas de la red pública de enseñanza, ubicadas en la zona urbana, con edades comprendidas entre los 13 y los 18 años.

Para la selección de la muestra se consideraron los siguientes criterios de inclusión: estar matriculado y asistir a la escuela los días en que se realizó la Recolección de datos; y, exclusión: presentar una limitación cognitiva que impidiera la participación en el estudio, discapacidad visual y/o auditiva que requiriera la necesidad de apoyo pedagógico especial para realizar las tareas escolares.

### Definición de la Muestra

Los adolescentes fueron seleccionados mediante muestreo intencional y el número se definió cuando se alcanzó la redundancia informativa, es decir, por saturación teórica^(15,16)^.

### Recolección de Datos

Para la recolección de datos, se adoptó la técnica del Grupo Focal (GF)^(17)^. Los GF se moderaron a partir de un guion de preguntas abiertas que suscitaron el debate, centrado en las estrategias de afrontamiento ante las adversidades ocasionadas por la pandemia de Covid-19, organizado en cuatro momentos: (a) presentación de los participantes; (b) dinámica para romper el hielo; (c) preguntas provocadoras centradas en la comprensión de las percepciones y experiencias de los adolescentes en la pandemia de COVID-19 (d) finalización.

Las preguntas provocadoras fueron las siguientes: ¿Es la COVID-19 una amenaza para los adolescentes? ¿Por qué la COVID-19 es una amenaza para la salud de los adolescentes? En su opinión, ¿en qué lugares es la COVID-19 una mayor amenaza y por qué? Pensando en todo el período de la pandemia, ¿cuáles fueron sus principales temores en relación con la enfermedad la COVID-19? ¿Por qué creían que podían tener síntomas graves? ¿Qué estrategias adoptaron para prevenir el coronavirus? Y, en su opinión, ¿cómo pueden los adolescentes ayudar a combatir el coronavirus? ¿Cuáles fueron las principales dificultades para hacer frente al coronavirus? ¿Por qué? ¿Cómo fue para ustedes la época de la COVID-19 en relación con la escuela, los amigos, el ocio, etc.? ¿Cómo se sintieron? ¿Cuáles fueron las consecuencias de este periodo para ustedes? ¿Qué te motiva a tomar medidas preventivas contra el coronavirus? Cuando lo necesitaron, tuvieron acceso a atención en salud y/o medicamentos, ¿cómo fue esa experiencia?

Los GF estaban compuestos por grupos pequeños/homogéneos, de entre 6 y 10 participantes, con una duración aproximada de una hora, grabados con equipo específico.

### Análisis y Tratamiento de los Datos

Los datos generados se organizaron siguiendo los siguientes pasos: transcripción íntegra, elaboración del inventario, que consiste en aislar los elementos presentes en las declaraciones y expresiones de los adolescentes, y generación del corpus textual.

Para ello, se utilizó el Análisis de Contenido Reflexivo, un método trans teórico y flexible orientado a la descripción y reducción de datos cualitativos manifiestos. Se trata de una técnica utilizada para identificar patrones en los significados superficiales explícitos de los datos cualitativos mediante el uso de una estructura jerárquica de estratos analíticos cuantificables denominados códigos, subcategorías y categorías. Cada estrato existe en un continuo de abstracción, siendo los códigos los más cercanos a los datos originales y las categorías los más abstractos. Durante cada etapa del proceso, la reflexividad se considera un recurso analítico valioso que es crucial para garantizar una descripción adecuada de los datos. La técnica está destinada a utilizarse como método de análisis de datos, y no como metodología; por lo tanto, puede integrarse con varios enfoques metodológicos y epistemológicos^(18)^.

Tras esta organización, el corpus textual se sometió a análisis en el software IRAMUTEQ (Interfaz de R para el Análisis Multidimensional de Textos y Cuestionarios). Para el análisis del corpus textual, se eligió la clasificación jerárquica descendente (CJD).

### Aspectos Éticos

Este estudio respetó las directrices éticas establecidas por la Resolución 466/2012 del Consejo Nacional de Salud^(19)^ y fue sometido y aprobado por el Comité de Ética en Investigación de la Universidad Federal de Piauí (CEP/UFPI), con el dictamen n.º 5.218.237.

Se preservó el anonimato de los participantes y los fragmentos de las declaraciones se identificaron con el código A. (de adolescente) seguido de una numeración identificativa del grupo (por ejemplo: A1, A2, A3, y así sucesivamente).

## RESULTADOS

Se realizaron seis GF (un GF por escuela), con una duración media de 51 minutos, en los que participaron 45 adolescentes, con una edad media de 15,88 (±1,43) años, la mayoría de sexo femenino (51,2 %), que se declararon de raza mestiza (48 %), de religión católica (57,1 %), solteras, sin pareja estable (53,7 %) y que vivían con sus padres (58,6 %).

El análisis del corpus textual generó 47 unidades de texto, 87 segmentos de texto, 556 formas distintas y 2655 ocurrencias de palabras. De acuerdo con el análisis jerárquico descendente, el material se organizó en seis clases semánticas, como se observa en la Figura 1.

**Figura 1:** Dendograma de las clases obtenidas a partir del corpus textual.

Las clases se dividieron entonces en tres ramificaciones (1, 2, 3) del *corpus* textual y se identificaron las proximidades semánticas entre ellas (Figura 1). Estas se reagruparon, generando tres categorías: percepción del riesgo y susceptibilidad (clases 1 y 3); gravedad y severidad de la COVID-19 (clases 2 y 4); percepción de la prevención y uso de medidas preventivas contra la infección por coronavirus (clases 5 y 6) (Cuadro 1).

**Cuadro 1:** Demostración de las clases y categorias – Picos, PI, Brasil, 2023.

Clase	Categorías
Clase 3	Percepción de riesgo y susceptibilidad a COVid-19
Clase 1	
Clase 4	Gravedad y severidad de COVID-19
Clase 2	
Clase 5	Prevención y medidas protectoras a la contaminación por coronavirus
Clase 6	

Fuente: Autores (2023).

### Categoría 1: Percepción del Riesgo y Susceptibilidad a COVID-19

Las clases 1 y 3 comprendieron el 38,2 % de los segmentos, lo que representa más de un tercio de las declaraciones de los adolescentes, que manifestaron inquietudes, sentimientos, miedos e ideas sobre la percepción del riesgo, la vulnerabilidad y la susceptibilidad a COVID-19, expresadas mediante palabras como amenaza, causa, muerte, enfermarse, transmitir.

*En el caso [la COVID-19], no [es solo un riesgo] para los adolescentes, es para todos, porque causa muertes. Es como una amenaza, visitar a personas mayores, no dar la mano [...] (A1).*

*El adolescente tiene [miedo] de morir, miedo de contraer la enfermedad [...] prevenirse [es una forma de] no contagiar a otras personas (A4).*

*[...] [existe el riesgo de que la COVID-19] se transmita a otras personas, como niños y personas mayores (A28).*

*[...] al principio pensé que no sería una amenaza para mí, y la gente decía que los que más se contagiaban eran los ancianos, los que estaban enfermos, pero luego empezaron a aparecer muchos casos de jóvenes y entonces me preocupé (A18).*

*[...] mantuve la distancia, usé cubrebocas, porque en mi casa hay personas mayores. Si me contagiaba, sería más peligroso para ellos (A2).*

*Hacíamos videollamadas con nuestros amigos (A7).*

### Categoría 2: Gravedad y Severidad de la COVID-19

Las clases 2 y 4 reunieron el 33,8 % de los segmentos textuales con similitud semántica en cuanto al reconocimiento de la morbilidad y mortalidad y la gravedad relacionadas con la infección por el nuevo coronavirus. Las declaraciones demostraron que el momento que vivieron fue difícil, por la necesidad de quedarse en casa y la intensificación de la adopción de medidas de higiene. Así, las palabras más frecuentes en las clases fueron: casa, alcohol, usar cubrebocas, aislamiento y miedo.

Los relatos de los adolescentes destacan la comprensión de la gravedad y severidad de la COVID-19, ejemplificada por el miedo a morir, la preocupación por la salud de los familiares, especialmente de las personas más vulnerables, y la necesidad de adoptar medidas de protección como el uso de cubrebocas y la mejora de los hábitos de higiene.

*[...] fue un tiempo que perdimos y, en esa época, no podíamos salir, tener contacto con otras personas, [porque] la enfermedad es una amenaza para los adolescentes. [Estar] con los amigos dejó de ser una realidad frecuente, y con las clases [en línea] dejamos de convivir, de ir a lugares donde había aglomeraciones por miedo (A13).*

*Los que sobreviven quedan con secuelas, la amenaza está relacionada con la enfermedad y la muerte [...] a veces usaba cubrebocas, [por] miedo a morir (A3).*

*[refiriéndose a COVID-19]. Como causó muchas muertes, tenía miedo de perder el gusto y el olfato, [...], miedo por mi hermana, que estaba embarazada y tuvo a mi sobrina durante la pandemia, ella era muy pequeña (A12).*

*Porque sufrían hasta morir [refiriéndose a las personas con COVID-19], usé cubrebocas, mejoré los aspectos de higiene y me quedé en casa, me vacuné. En cuanto a sentirse solo, creo que da igual [...] (A19).*

*Cuando contraje COVID, lloraba casi todo el tiempo por miedo a que ocurriera lo peor (A22).*

*Porque hoy en día existe la vacuna y prácticamente ya no hay virus graves, (miedo) de contagiar a mis padres, usé cubrebocas y gel desinfectante [...] (A 24).*

*No tuve miedo, ni me contagié, pero usé cubrebocas y sigo usando alcohol gel hasta hoy, mantuve la distancia a veces, evité las aglomeraciones y los lugares cerrados (A. 46).*

La percepción de la gravedad y la severidad del momento vivido provocó en los adolescentes la asimilación de una nueva realidad, en la forma de vivir y relacionarse, así como la adaptación al contexto de crisis.

*[refiriéndose a la COVID-19]. No creía que fuera a afectar a los jóvenes, porque tienen una inmunidad alta, hasta el punto de llegar a mi casa y acabar llevándose a mi abuela, era una época en la que no había vacuna, nada (A14).*

*Porque todo el mundo estaba socializando, era época de carnaval y entonces todo tuvo que parar (A11).*

*[...] de hacer amigos, luego la mayoría se volvió tímida porque nos acostumbramos mucho a estar en casa (A13).*

*La COVID-19 afectó a toda nuestra generación y cambió por completo la forma en que nos cuidamos, al principio tomó a todos por sorpresa, nadie lo esperaba y de repente mucha gente comenzó a morir y los síntomas se agravaron (A. 17).*

### Categoría 3: Prevención y Medidas de Protección Contra el Contagio por Coronavirus

Esta categoría de análisis abarca las clases 5 y 6, y representa el 27,9 % del corpus textual. Entre las palabras más frecuentes de las clases se encuentran: mascarilla, alcohol, gel, evitar, salud, inmunidad, mano, higiene, aglomeración, distanciamiento, aislamiento social.

Las declaraciones de los participantes ilustran las estrategias de prevención de la infección por coronavirus que adoptaron los adolescentes durante la pandemia para romper la cadena de transmisión, en especial el distanciamiento social.

*Higiene, sobre todo cuando vienes de la calle, la vitamina C ayuda a la inmunidad, usar mascarilla y lavarse las manos (A. 36).*

*[...] usar cubrebocas, lavarse las manos porque nuestras manos tocan muchos lugares [...] (A. 37).*

*Hay formas de prevenirse, como quedarse en casa, porque es una enfermedad que mata. En las escuelas, [refiriéndose a eventos sociales] fiestas, usé cubrebocas y sigo usando alcohol gel, mantuve la distancia a veces; evité las aglomeraciones y los lugares cerrados (A.43, A.47).*

*Evitar salir de casa porque los adolescentes tienen mayor inmunidad, por lo que si se protegen no contagiarán a otras personas, evitar el contacto con personas mayores (A. 36).*

*Fue difícil estar lejos de las personas que queremos, de los amigos (A. 34).*

Los adolescentes señalaron la inmunidad y el acceso a los servicios de salud como medidas preventivas relacionadas con el control, la prevención y la reducción de la transmisión del coronavirus. La mayoría afirmó no haber necesitado atención médica durante el periodo, pero, cuando fue necesario, consiguieron atención.

*[...] recibí atención médica. Necesitaba hacerme unas pruebas y las conseguí, pero la prioridad era la COVID (A. 38).*

## DISCUSIÓN

Los resultados de este estudio evidencian que los adolescentes desarrollaron una percepción ampliada sobre la pandemia de COVID-19, organizada en tres ejes analíticos centrales: percepción del riesgo y la susceptibilidad, gravedad y severidad de la enfermedad, y percepción de la prevención con la adopción de medidas preventivas. Estos resultados refuerzan las evidencias de la literatura que señalan que, aunque los adolescentes suelen describirse como un grupo con menor percepción del riesgo individual, en contextos de crisis sanitaria esta población demuestra capacidad para comprender las amenazas para la salud y ajustar sus comportamientos ante los riesgos colectivos^(20,21)^.

La percepción del riesgo y la susceptibilidad, identificada en las clases 1 y 3, revela que los adolescentes reconocieron la posibilidad de infección tanto para ellos mismos como para las personas cercanas, especialmente los familiares. Este reconocimiento es un componente central del Modelo de Creencias en Salud, según el cual la percepción de la susceptibilidad influye directamente en la adopción de comportamientos protectores^(13)^. Los estudios realizados durante la pandemia indican que los adolescentes que perciben un mayor riesgo personal y social tienden a adherirse de manera más consistente a las recomendaciones sanitarias, como el distanciamiento social y el uso de cubrebocas, lo que corrobora los hallazgos de este estudio^(22,23)^.

El modelo de creencias en salud ha demostrado ser importante para comprender cómo los adolescentes perciben y responden a una crisis emergente, como la pandemia de COVID-19, lo que sugiere que la percepción de la vulnerabilidad y la gravedad de la enfermedad, junto con la creencia en la eficacia de las medidas preventivas, influyen en el comportamiento de salud de las personas. En el caso de los adolescentes, la percepción de la gravedad de la COVID-19 y la creencia en la eficacia de las prácticas recomendadas para contener la pandemia fueron determinantes para la adopción de comportamientos preventivos durante este periodo^(24,25)^.

En este sentido, se debe reforzar el cuidado de la salud entre los adolescentes, dándoles la oportunidad de comprender la percepción del riesgo, la susceptibilidad, la gravedad y la severidad del comportamiento y/o la exposición a riesgos para la salud para tomar decisiones sobre medidas preventivas.

En cuanto a la gravedad y severidad de COVID-19, representadas por las clases 2 y 4, las declaraciones demuestran que los adolescentes comprendieron la pandemia como un evento de gran impacto, capaz de modificar rutinas, relaciones sociales y proyectos de vida. Esta percepción está en consonancia con el constructo de gravedad percibida del modelo de creencias en salud, que se refiere a la evaluación de las consecuencias físicas, sociales y emocionales de una enfermedad^(13)^. Aunque COVID-19 presenta una menor letalidad entre los adolescentes, los estudios indican que la percepción de la gravedad estuvo fuertemente asociada al miedo a transmitir el virus a los familiares, especialmente a las personas mayores, y a las repercusiones psicosociales del aislamiento social^(6,26)^.

La categoría relacionada con la percepción de la prevención y el uso de medidas preventivas (clases 5 y 6) evidencia que los adolescentes asimilaron información sobre estrategias de control de la transmisión del coronavirus, siendo el distanciamiento social, la higiene de las manos y el uso de cubrebocas las prácticas más mencionadas. Estos hallazgos sugieren la eficacia de las acciones de comunicación en salud desarrolladas en el país durante la pandemia, en consonancia con las directrices del Ministerio de Salud y la Organización Mundial de la Salud^(27,28)^. Además, la mención de la inmunidad y el acceso a los servicios de salud como medidas preventivas demuestra una comprensión ampliada de la atención sanitaria, que implica tanto comportamientos individuales como la organización de los sistemas de salud.

En el contexto brasileño, estos resultados dialogan directamente con las políticas públicas de salud y educación, especialmente con los principios del Sistema Único de Salud (SUS), como la universalidad, la integralidad y la equidad, y con las directrices de la Política Nacional de Promoción de la Salud (PNPS), que enfatiza el protagonismo de los sujetos y el fortalecimiento de la autonomía para el cuidado^(29)^. Además, la articulación entre salud y educación^(30)^, prevista en el Programa Salud en la Escuela (PSE), resulta fundamental para el desarrollo de acciones educativas dirigidas a los adolescentes, sobre todo en situaciones de emergencia sanitaria, en las que la escuela y los entornos digitales se convierten en espacios estratégicos para la difusión de información fiable.

Cabe destacar que la mayoría de los adolescentes informaron no haber necesitado atención en salud durante el período pandémico, pero aquellos que buscaron asistencia lograron acceder a los servicios. Este dato puede reflejar la percepción de la eficacia del SUS, a pesar de las dificultades impuestas por la pandemia, y actuar como factor de protección al reforzar la confianza en el sistema de salud.

En general, los resultados indican que la pandemia de COVID-19 funcionó como un evento catalizador para el desarrollo de la conciencia en salud entre los adolescentes, al ponerlos en estado de alerta y favorecer la comprensión del riesgo individual y colectivo^(24,25)^. Estos hallazgos refuerzan la necesidad de reconocer a los adolescentes como sujetos activos en las políticas públicas de salud, capaces de comprender información compleja y de participar de manera responsable en las estrategias de prevención. Así, invertir en acciones educativas basadas en modelos teóricos, como el modelo de creencias en salud, integradas en las políticas del Sistema Único de Salud (SUS) y del sector educativo, puede fortalecer la promoción de la salud y la preparación de esta población para afrontar futuras crisis sanitarias.

Cabe señalar que los datos presentados en este estudio se basaron exclusivamente en respuestas autoinformadas de adolescentes que pueden estar sujetas a sesgos de deseabilidad social, limitadas a un contexto geográfico y cultural específico.

Sin embargo, los hallazgos pueden ayudar a comprender cómo los adolescentes enfrentaron este período, así como los posibles impactos para la salud y el desarrollo de este público, fundamentando acciones de salud dirigidas a mitigar estos impactos.

## CONCLUSIÓN

El estudio demostró cómo la pandemia de COVID-19 afectó la vida de los adolescentes, que se acompañó de percepciones de riesgo, susceptibilidad y gravedad distintas y complejas. En particular, la percepción del riesgo y la gravedad de la crisis sanitaria contribuyó a que los adolescentes adoptaran comportamientos de autoprotección tanto para ellos mismos como para sus familias. Cabe destacar las manifestaciones de sentimientos de miedo, ansiedad, aislamiento y dificultades que pueden tener repercusiones en la salud mental y las interacciones sociales de los adolescentes en el período pospandémico.

Los hallazgos refuerzan el papel estratégico de la enfermería en la mediación entre la información, la percepción del riesgo y la adopción de comportamientos preventivos entre los adolescentes. En la práctica asistencial, los enfermeros de la APS y del PSE pueden desarrollar acciones educativas basadas en el modelo de creencias en salud, tales como: círculos de conversación que exploren la percepción de la susceptibilidad y la gravedad de las enfermedades transmisibles; estrategias de asesoramiento breve para adolescentes, incorporando ejemplos de la vida cotidiana escolar y familiar; uso de lenguaje accesible y recursos digitales para reforzar los beneficios percibidos de las medidas preventivas.

El estudio recomienda que el modelo de creencias en salud ayude a los adolescentes a estar preparados para adoptar comportamientos preventivos, ya que se basa en la comprensión del riesgo personal y de su entorno, de la susceptibilidad y gravedad de la enfermedad y/o de la exposición al riesgo para la salud, ya sea a nivel individual o colectivo.

En el contexto escolar, se sugiere la inclusión transversal de temas como la prevención de enfermedades transmisibles y el pensamiento crítico sobre la información en salud, fortaleciendo la alfabetización en salud de los adolescentes. La articulación entre salud y educación puede contribuir a respuestas más consistentes ante futuras emergencias sanitarias.

En cuanto a las políticas públicas, los resultados indican que deben tenerse en cuenta los determinantes psicosociales que influyen en el comportamiento de los adolescentes, como las creencias, los valores, las normas sociales y el acceso a los servicios de salud.

Para futuras investigaciones, se recomienda el desarrollo de estudios longitudinales que hagan un seguimiento del mantenimiento de los comportamientos preventivos a lo largo del tiempo, análisis comparativos entre diferentes regiones y realidades socioeconómicas, investigaciones de intervención realizadas por enfermeras, evaluando estrategias educativas basadas en el modelo de creencias en salud, y estudios que integren métodos cualitativos y cuantitativos para profundizar en la comprensión de las creencias y actitudes de los adolescentes frente a las crisis sanitarias.

## Data Availability

El conjunto de datos que respaldan las conclusiones de este estudio está disponible previa solicitud al autor correspondiente.
